# I-SWOT as instrument to individually optimize therapy of thoracoabdominal aortic aneurysms: Effective, norm-compliant and meeting the needs

**DOI:** 10.1007/s00772-017-0245-z

**Published:** 2017-02-07

**Authors:** A. Sachweh, Y. von Kodolitsch, T. Kölbel, A. Larena-Avellaneda, S. Wipper, A. M. Bernhardt, E. Girdauskas, C. Detter, H. Reichenspurner, C. R. Blankart, E. S. Debus

**Affiliations:** 1 Department of General and Interventional Cardiology, German Aortic Center Hamburg (DAZH), University Heart Center Hamburg, University Medical Center Hamburg Eppendorf, Martinistraße 52, 20246 Hamburg, Germany; 2 Department of Vascular Medicine, German Aortic Center Hamburg (DAZH), University Heart Center Hamburg, University Medical Center Hamburg Eppendorf, Hamburg, Germany; 3 Department of Cardiovascular Surgery, German Aortic Center Hamburg (DAZH), University Heart Center Hamburg, University Medical Center Hamburg Eppendorf, Hamburg, Germany; 4 Hamburg Center for Health Economics, University of Hamburg, Hamburg, Germany

**Keywords:** Medical decision making, Evidence based medicine, Thoracoabdominal aortic aneurysm, Individualized decision, Optimization, Medizinische Entscheidungsfindung, Evidenzbasierte Medizin, Individualisierte Entscheidung, Thorakoabdominales Aortenaneurysma, Optimierung

## Abstract

**Background:**

Guidelines summarize medical evidence, they identify the most efficient therapy under study conditions and recommend this therapy for use. The physician now has the challenge to translate a therapy that is efficient under laboratory conditions to a patient who is an individual person. To accomplish this task the physician has to make sure that (I) the ideal typical therapy is applicable and effective in this individual patient taking the special features into consideration, that (II) therapy is compliant with the norm including guidelines, laws and ethical requirements (conformity) and that (III) the therapy meets the patient’s needs.

**Objective:**

How can physicians together with the patients translate the medical evidence into an individually optimized therapy?

**Material and methods:**

At the German Aortic Center in Hamburg we use I‑SWOT as an instrument to identify such individually optimized therapy. With I‑SWOT, we present an instrument with which we have developed an (I) efficient, (II) conform and (III) needs-oriented therapeutic strategy for individual patients.

**Results:**

I-SWOT cross-tabulates strengths (S) and weaknesses (W) related to therapy with opportunities (O) and threats (T) related to individual patients. This I‑SWOT matrix identifies four fundamental types of strategy, which comprise “SO” maximizing strengths and opportunities, “WT” minimizing weaknesses and threats, “WO” minimizing weaknesses and maximizing opportunities and “ST” maximizing strengths and minimizing threats. We discuss the case of a patient with asymptomatic thoracoabdominal aneurysm to show how I‑SWOT is used to identify an individually optimized therapy strategy.

## Zusammenfassung

**Hintergrund:**

Leitlinien fassen medizinische Evidenz zusammen, identifizieren die unter Studienbedingungen effektivste Therapie und empfehlen diese zur Anwendung. Der Arzt steht vor der Herausforderung, eine unter Studienbedingungen effektive Therapie auf individuelle Patienten anzuwenden. Dazu muss er sicherstellen, dass (I) die idealtypisch effektive Therapie auch unter Berücksichtigung der individuellen Besonderheiten seines Patienten einsetzbar und wirksam ist (Effizienz), dass (II) sein Vorgehen konform geht mit Leitlinien, Gesetzen- und ethischen Forderungen (Konformität), und dass (III) die Therapie den Bedürfnissen des Patienten gerecht wird (Bedürfnisorientierung).

**Fragestellung:**

Wie können Ärzte zusammen mit ihren Patienten einen Weg zu einer individuellen optimalen Therapie finden?

**Material und Methoden:**

Mit I‑SWOT stellen wir ein Instrument vor, mit dessen Hilfe wir am Deutschen Aortenzentrum Hamburg eine (I) effiziente, (II) konforme und (III) bedürfnisorientierte Therapiestrategie für den individuellen Patienten entwickeln.

**Ergebnisse:**

I-SWOT besteht aus einer Vierfeldertafel, in deren Kopfzeile wir die Stärken („strengths“) und Schwächen („weaknesses“) verschiedener Therapieoptionen auflisten. In der Vorspalte der Tafel listen wir die Chancen („opportunities“) und Gefahren („threats“) auf, die ihre Ursachen in individuellen Charakteristika des Patienten haben. Es resultiert eine Vierfeldermatrix, die vier Grundvarianten der Strategie darstellt. Erstens: „SO“ als Maximierung von Stärken und Chancen. Zweitens: „WT“ als Minimierung von Schwächen und Gefahren. Drittens: „WO“ als Minimierung von Schwächen und Maximierung von Chancen. Viertens: „ST“ als Maximierung von Stärken und Minimierung von Gefahren. Die vier Typen der Strategie bilden die Grundvarianten einer individualisierten medizinischen Strategie. Anhand des Fallbeispiels eines Patienten mit asymptomatischen thorakoabdominalen Aortenaneurysma illustrieren wir, wie wir mittels I‑SWOT eine individuell optimale therapeutische Strategie entwickeln.

## Background

**Fig. 1:**
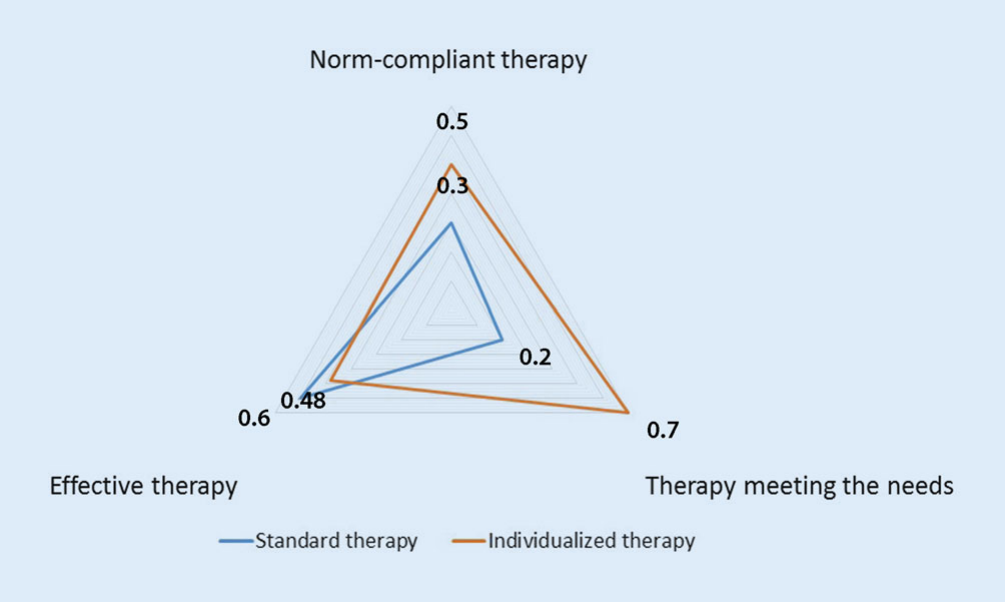
Two forms of guideline-based therapy. Firstly, the evidence-based medicine uniform therapy is used to treat every patient according to the same medical standard and risks technical and ethical treatment failure [1]. Secondly, an individualized medical strategy attempts to achieve optimum adaptation of medical standards to individual patients using instruments such as I‑SWOT. Measured as the area of both triangles, therapy success in the three dimensions effectiveness, norm compliance and needs-oriented is greater in the individualized medical therapy (*red triangle*) than in the evidence-based medicine uniform approach (*blue triangle*)

Guidelines summarize medical evidence, they identify the most efficient therapy under study conditions and recommend this therapy for use. The physician now has the challenge to translate a therapy that is efficient under laboratory conditions to a patient who is an individual person. To accomplish this task, the physician has to make sure that (i) the ideal therapy is applicable and effective in this individual patient taking the special features into consideration, that (ii) therapy is compliant with the norm including guidelines, laws and ethical requirements (conformity) and that (iii) the therapy meets the patient’s emotional and psychological needs. Together with the patient, the physician has to find the best way for an individualized optimal therapy. With I‑SWOT we introduce an instrument which enables us to develop individualized medical therapies for individual patients at the German Aortic Center Hamburg (DAZH) that are efficient compliant and needs-oriented (Table 1; Fig. 1).

**Table 1:** Definition of some terms of individualized medical strategy (IMS)

Efficiency	The physical success of therapy under controlled study conditions
Effectiveness	The physical success of therapy for an individual patient under real world conditions
Conformity	The compliance of a therapy according to guidelines, laws and ethical issues
Needs orientation	A therapy that takes into account the (i) physical (ii) social and (iii) emotional needs of the patient
Optimization	Individual therapy success in three dimensions, effectiveness, conformity and needs orientation aimed at goal optimization, because maximum success in all three dimensions simultaneously is hardly possible
Strengths (S)^a^	The characteristics of a therapeutic option for maximizing the success of treatment in a (i) physical (ii) normative and (iii) emotional dimension
Weaknesses (W)^a^	The characteristics of a therapeutic option that are contrary to maximizing the success of treatment in a (i) physical (ii) normative and (iii) emotional dimension
Opportunities (O)^a^	The characteristics of an individual patient, which are promotive for maximizing the success of therapy in a (i) physical (ii) normative and (iii) emotional dimension
Threats (T)^a^	The characteristics of an individual patient, which are obstructive for maximizing the success of therapy in a (i) physical (ii) normative and (iii) emotional dimension

^a^These points are evaluated by the physician

## What does individual therapy success mean?

The statement “we do therapy according to guidelines” may stand for two fundamentally different ideas of therapy (Fig. 1). First, this statement may express the idea of therapy as a one size fits all way of doing standardized medicine irrespective of individuality; however, therapy that ignores the physical conditions of the individual patient risks technical treatment failure. For example, an aortic stent graft may be the best therapeutic option according to the guidelines but such therapy may not be feasible because of a specific aortic anatomy in an individual patient. Moreover, standard therapy may ignore patient’s autonomic will and values and it may thereby result in ethical failure. Finally, therapy according to uniform standards may lack the patient’s motivational support and therefore result in emotional failure of therapy. Alternatively, we interpret “doing therapy according to guidelines” as the task to fit standard recommendations to the specific conditions of individual patients to maximize therapeutic success. Maximizing therapeutic success requires maximizing success in three dimensions comprising (i) biology of the disease and patient’s physical make-up, (ii) norms with conformity of therapy with patient’s autonomy, with medical guidelines, and laws, and (iii) emotions including the patient’s motivational support of therapy. Therefore, individualized therapeutic success results from success in these three therapeutic dimensions (i) as effective therapy, by attaining the physical goals of therapy, such as elimination of the aneurysm (“do therapy right”), (ii) as norm-compliant therapy by attaining normative objectives, such as compliance with guidelines, laws and ethical values (“do the right therapy”) and (iii) as needs-oriented therapy by achieving emotional goals, such as the patient’s full identification with therapeutic efforts (“therapy feels good”) [1].

**Fig. 2:**
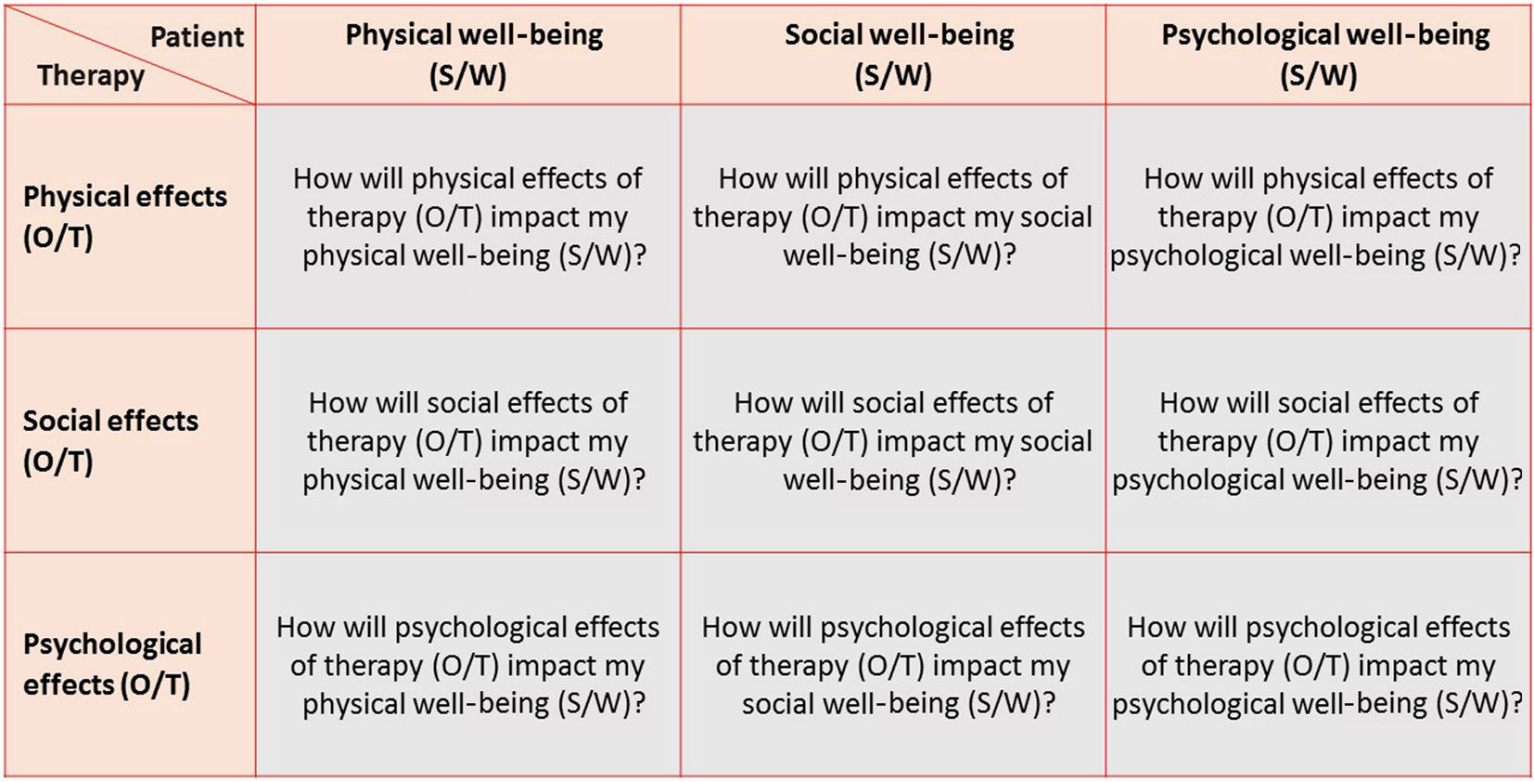
3 × 3 questions to optimize therapy from the patient’s point of view

**Fig. 3:**
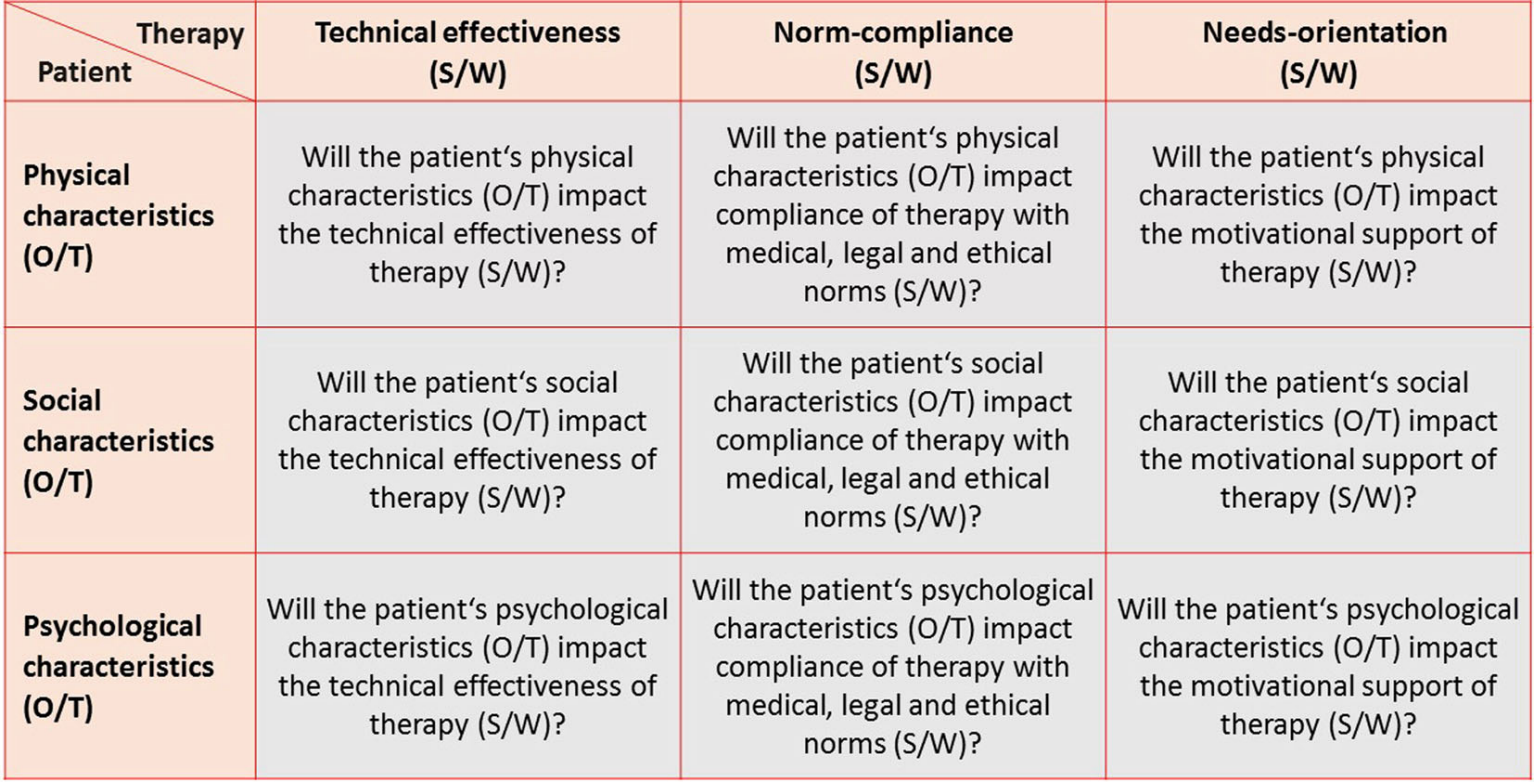
3 × 3 questions to maximize therapy from the physician’s point of view

To maximize therapeutic success, we need to consider all three dimensions. To this end the patient asks himself other questions than the physician does. The patient may inquire which (I) physical, (ii) social and (iii) psychological consequences of therapy he would consider as an opportunity (O) or as a threat (T) for his (i) psychological, (ii) normative and (iii) emotional well-being (Fig. 2). In contrast the physician usually asks which (i) physical, (ii) social and (iii) psychological characteristics of the patient may be an opportunity (O) or a threat (T) for the (i) physical, (ii) normative and (iii) the emotional success of the therapy (Fig. 3).

## Why do we advocate an individually optimized therapy?

Vascular medicine is predominantly concerned with treating old and very old patients with serious comorbidities; therefore, the question to be answered is how these patients can live with dignity during the last phases of their lives and whether they will be able to tolerate and accept drastic consequences of a treatment. Dignity and intensity of life, independence and mobility are the key issues, which are quite essential. Hence, decision making may be very difficult and absolutely requires individual solutions. Furthermore, for one patient an amputation can be the relief of unquenchable pain, for another patient it may be the beginning of a new mobility with a prosthesis but also an irreversible step towards dependency and surrender.

The compliance with patient-related priorities may even push the compliance with guideline-based treatments into the background, as can be concluded from various examples of all domains of medicine. For example, a patient suffering from gastric cancer needs a gastrectomy and a jejunal substitute stomach to achieve R0 resection according to the guidelines; however, the loss of quality of life by massive weight loss and significant restriction of food intake may probably be an intolerable result of this intervention so that the long-term survival (with low quality of life) may be pushed into the background. Or a patient with ulcerative colitis without complications, whose only curative chance is the guideline-based therapy of a proctocolectomy; however, this means the need for a radical change in life style and probably occupational disability by expectedly persistent diarrhea many times a day. All this could be a strong argument against a prophylactic intervention which aims exclusively at protecting the patient from the formation of a carcinoma of the colon.

Many examples from vascular medicine can be cited: the data from the literature show that the natural clinical course of thoracoabdominal aortic aneurysm (TAAA) has not yet been extensively researched; therefore, we only know grossly at which diameter the risk of rupture is increased and how aneurysms develop in the natural history of disease [2]. Generally, a diameter of 5.5 cm is considered as an indication for invasive surgery [3]. Since the introduction of endovascular techniques, the number of treatments has increased by the factor of ten, despite the high mortality and risk of paraplegia. Should we really offer this treatment to a 70-year-old patient who has an asymptomatic thoracoabdominal aneurysm, 5.5 cm in size, enjoying good quality of life without limitations, with knowledge of all the risks? What will it really mean to these patients to gain 10 years in a wheelchair if a rupture will perhaps occur after 8 years? We do not know. Can we really make this decision without consulting the patient? According to the idea of I‑SWOT, the patient’s opinion is at least of equal value as that of the physician.

## What is the idea of I‑SWOT?

Medical standards require translation into individual characteristics of patients. Currently, there are no instruments that provide an easy to use but formal and systematic method to perform this translation. I‑SWOT is an instrument that performs this task. Here, we briefly outline how we use I‑SWOT at the German Aortic Center Hamburg (DAZH). We presented a detailed description of I‑SWOT and its theoretical basis elsewhere [4]. SWOT analysis is a classical instrument for strategic planning. We can find examples for its successful practice in the martial arts [5], management and project planning [6]. SWOT attains maximum success by the comparison of own strengths (S) and weaknesses (W) with environmental opportunities (O) and threats (T). From this comparison, the decision maker chooses a strategic option for further action. You may consider a boxer who compensates his own “weaknesses”, exploiting his “strengths”, and who escapes his opponent’s “threats” exploiting “opportunities” arising from his opponent to win victory. In a similar way, the physician seeks to exploit “strengths” and “weaknesses” of different therapeutic options balancing these with patient-associated “opportunities” and “threats” to maximize therapeutic success.

We show that I‑SWOT is a method which can be used by healthcare professionals as a standardized method to optimize the therapeutic outcome based on medical guidelines. In this article we choose the doctor’s perspective, who performs I‑SWOT to achieve an optimum therapeutic strategy. Alternatively, the patient will be able to conduct his own I‑SWOT analysis by using information and knowledge of the therapeutic options to maximize his or her benefits from treatment. The optimization of patient’s therapy could be achieved by matching both points of view in a doctor–patient dialogue, which is the major method to approach an optimal therapeutic decision [1].

## Four types of strategies for optimized individual therapy success

**Fig. 4:**
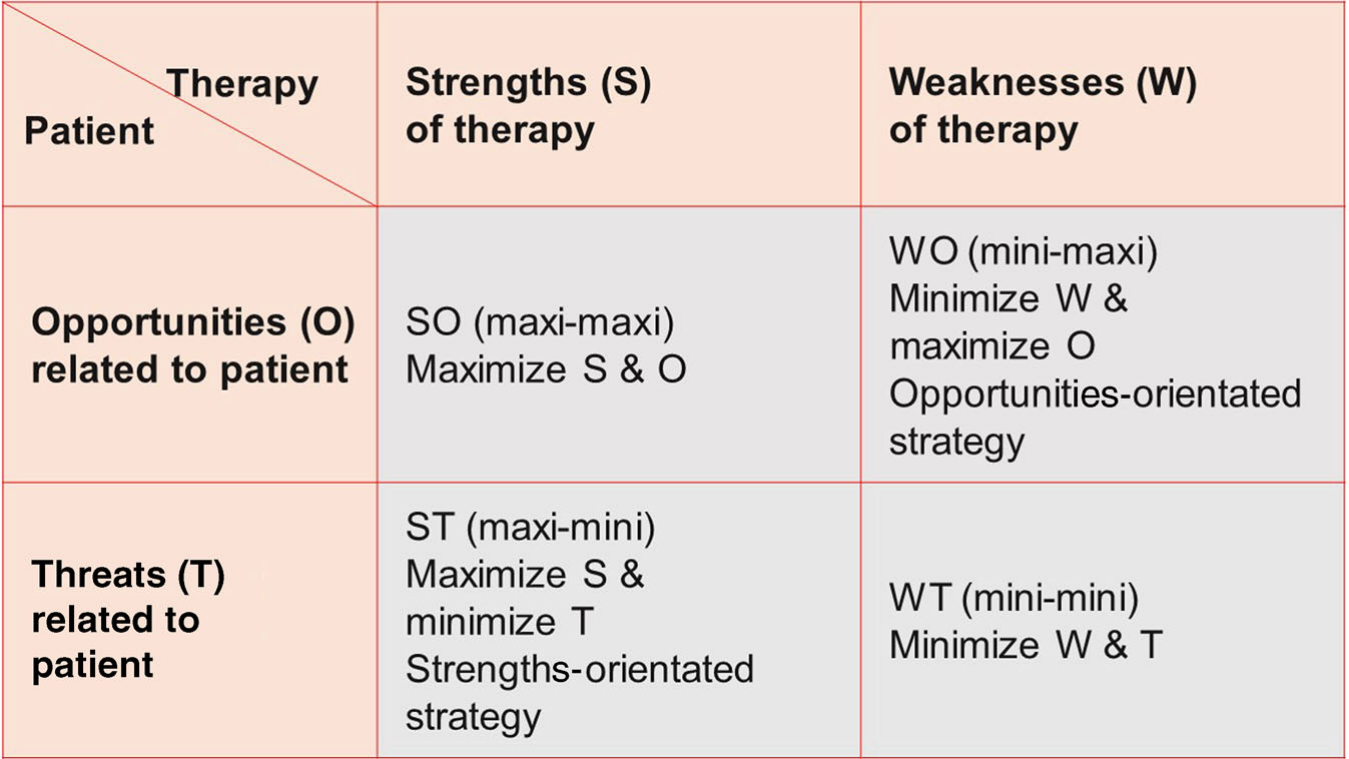
I-SWOT matrix for the evaluation of therapy-related strengths and weaknesses in relation to the patient-related opportunities and threats for the success of treatment

We perform I‑SWOT as a 4‑field matrix in which strengths (S) and weaknesses (W) of various therapeutic options are listed in the column headings. In the left column of the table we fill in opportunities (O) and threats (T) related to the individual characteristics of the patient. The result is a matrix that represents the four classical types of strategies (Fig. 4):

The SO strategy maximizes both the therapeutic strengths and patient-related opportunities (maxi-maxi strategy). This strategy is typically assumed in situations with abundant availability of both therapeutic strengths (S) and patient’s opportunities (O). The vulnerability of this type of strategy lies in the overestimation of the strengths of novel or complex techniques, and overconfidence in patient-related opportunities.

The WT strategy minimizes both therapeutic weaknesses and patient-related threats (mini-mini strategy). Such a strategy might be chosen in precarious situations, when therapeutic options are sparse and threats are mounting. Physicians should strive to avoid such unfortunate situations and seek other strategic options; however, a WT strategy may be useful to gain time to improve the initial situation, e. g by stabilizing a patient, to organize advanced therapy or to transfer the patient to specialized centers with better therapeutic options.

The WO strategy minimizes weaknesses and maximizes opportunities (mini-maxi or opportunity-oriented strategy). Physicians prefer to choose this strategy in situations where therapeutic weaknesses are evident, while external opportunities are appealing. A physician could, for example, organize a transport by helicopter to the next cardiac surgery medical center for patients with acute type A aortic dissection; however, physicians and hospitals should try to reduce their own therapeutic weaknesses and develop their strengths.

The ST strategy maximizes own strengths and minimizes patient’s threats (maxi-mini or strength-oriented strategy). Physicians may choose this strategy in the face of desperate disease constellations, where maximizing own therapeutic strengths may be the only way to overcome substantial patient’s threats. Physicians may focus on their own intuition and their surgical and medical capabilities to rescue patients despite threatening conditions, especially when established or well-defined therapeutic options are not available. The challenge of a strength-oriented approach is to always protect oneself against undue self-reliance.

## The four steps of I‑SWOT

I-SWOT is a technique which may be intuitively applied: for example, a high-performance athlete weighs his abilities against those of his competitors without using a strategy matrix. Similarly, clinical decision-making is usually done without explicit use of the decision-making techniques. In our case conferences at the DAZH we usually apply I‑SWOT without applying formalized step by step techniques. The following illustration explains the procedure in four steps:

### 1. Define the goal of therapy and the spectrum of evidence-based therapeutic options.

In this step we analyze the clinical problem of a patient with aortic disease to identify the therapeutic goal. Examples of such goals are protection against aortic rupture, prevention of false lumen expansion in chronic aortic dissection type Stanford B, or stabilization of contained rupture of an abdominal aortic aneurysm. First, we describe the spectrum of evidence-based therapeutic options for a given therapeutic goal. Let us consider a patient with asymptomatic TAAA where we identify protection against aortic rupture as therapeutic goal. Next, we identify evidence-based consensus recommendations for five distinct therapeutic options as follows [7–10]: conservative therapy (A) implying medicinal therapy with angiotensin II receptor blockers (ARB), angiotensin-converting enzyme inhibitors (ACEI), beta adrenergic blockers (BAB) or calcium channel blockers (CCB), therapy for obstructive sleep apnea syndrome (OSAS), and behavioral modification with avoidance of Valsalva maneuvers or isometric muscle activities. Another option is endovascular therapy using so-called chimney, snorkel, periscope, or sandwich techniques (B) [7]. Complete endovascular repair with fenestrated and branched endovascular stent grafts (C) is another therapeutic option. Alternatively, hybrid procedures with visceral vessel debranching and stent grafts for the aorta (D), or open surgical repair with complete prosthetic graft replacement of TAAA (E) may be used to treat TAAA (Table 2; [11]).

**Table 2:** Protection against rupture by asymptomatic TAAA: strengths-weaknesses matrix for elective therapeutic options

Therapeutic options		Strengths (S)	Weakness (W)
(A)	Conservative therapy	No restrictions for quality of life (S1) Outpatient treatment (S2)	No reliable protection against aortic rupture (W1) Drug intolerance (W2)
(B)	Endovascular parallel techniques (chimney graft)	Prosthetic exclusion of TAAA (S3) No thoracotomy (S4) No laparotomy (S5) Option for patients with high risk for open surgical repair (S6) Immediate availability of prosthesis (S7) Good 30-day survival and immediate outcome (S8) Short stay in the ICU, hospital and rehabilitation (S9)	Risk of endoleaks, progression of aneurysm (W3) Bypass or transposition of the left subclavian artery may be needed (W4) Unsuitable vascular anatomy (W5) Endoprosthesis-related risks (W6) Procedural risks of endoprosthesis (W7) Risk of organ ischemia (W8) Off-label therapy (W9) Result depends on the surgeon (W10) Long-term results unknown (W11) Frequent check-ups with CT imaging (W12) Secondary procedures may carry increased risk (W13)
(C)	Complete endovascular surgery (fenestrated and branched stent grafts)	Prosthetic exclusion of TAAA (S3) No thoracotomy (S4) No laparotomy (S5) Option for patients with high risk for open surgical repair (S6) Good 30-day survival and immediate outcome (S8) Short stay in the ICU, hospital and rehabilitation (S9) Good results at the DAZH (S10)	Risk of endoleaks, progression of aneurysm (W3) Bypass or transposition of the left subclavian artery may be needed (W4) Unsuitable vascular anatomy (W5) Endoprosthesis-related risks (W6) Procedural risks of endoprosthesis (W7) Result depends on the surgeon (W10) Long-term results unknown (W11) Frequent check-ups with CT imaging (W12) Long planning period and delivery time for endoprosthesis (W14) Laparatomy (W15)
(D)	Hybrid operation (visceral debranching and aortic stent graft)	Prosthetic exclusion of TAAA (S3) No thoracotomy (S4) Option for patients with high risk for open surgical repair (S6) Immediate availability of the prosthesis (S7) Practicable despite unfavorable landing zones (S11)	Risk of endoleaks, progression of aneurysm (W3) Bypass or transposition of the left subclavian artery may be needed (W4) Endoprosthesis-related risks (W6) Procedural risks of endoprosthesis (W7) Procedural risks of laparotomy (W8) Result depends on the surgeon (W10) Long-term results unknown (W11) Frequent check-ups with CT imaging (W12) Laparotomy essential (W15) Aortic rupture in the interval before reoperation (W16) Longer ICU stay, hospital stay and rehabilitation (W17)
(E)	Open TAAA operation	Complete resection of TAAA (S12) Reimplantation of segmental arteries possible (S13) Operation applicable for all aortic pathologies (S14) Good results at the DAZH (S10) First choice therapy for Marfan syndrome (15) Long-term results >20 years (S16)	Results depend on the surgeon (W10) Longer ICU stay, hospital stay and rehabilitation (W17) Pseudo- or patch aneurysm, prosthetic infection (W18) Thoracotomy and laparotomy (W19) Not possible in high-risk cases for open surgery (W20) Specialized anesthesia, neuro-monitoring (W21)

*CIN* contrast-induced nephropathy, *DAZH* German Aortic Center of Hamburg, *ICU* intensive care unit, *TAAA* thoracoabdominal aortic aneurysm, *TAAA I, II, III, IV, and V* refer to the Crawford classification of TAAA

Explanatory notes*: W4* Bypass or left subclavian artery transposition according to the guidelines [13]; *W5* Hostile aortic landing zone (landing zone <2 cm, massive aortic calcification or thrombosis, “gothic aortic arch” anatomy), unsuitable aortic anatomy (aortic kinking, narrow vessel caliber, unfavourable aortic anatomy of outflow vessels), unsuitable access to vessels (calcification, kinking, simultaneous access from multiple vessels); *W6* Stent migration, stent collapse (risk factors are small diameter of aortic landing zone, aggressive oversizing, narrow aortic curvature with “bird-beaking” configuration); *W7* High radiation exposure, high contrast load with increased risk of allergic reactions, complications due to complex arterial access techniques, contrast**-**induced nephropathy (CIN) and dialysis with subsequent risk factors for CIN: diabetes mellitus, age > 75 years periprocedural volume depletion, heart failure, cirrhosis or nephrosis, arterial hypertension, proteinuria, pretreatment with nonsteroidal anti-inflammatory drugs (NSAIDs), initial intra-arterial injection of contrast medium [7]; *W8* Risk of organ ischemia (stroke, paraparesis, paraplegia especially with endograft >15 cm in length), visceral ischemia, renal artery infarctions; *W19* Thoracotomy with aortic clamping, extracorporeal circulation, and unilateral pulmonary ventilation; *W20* Patients who generally fulfil at least three of the following criteria: chronic arterial hypertension, chronic obstructive pulmonary disease with FEV1 < 1.0, coronary heart disease with myocardial infarction, stenting or aortocoronary bypass, heart failure with LVEF < 35% and >NYHA I, chronic renal failure with creatinine 1.2 mg/dl, American Society of Anesthesiologists score (ASA) ≥ 3, pre-existing aortic operation with thoracotomy or infrarenal aortic prosthetic grafts [14]

### 2. Identify the strengths and weaknesses of each therapeutic option (SW matrix).

This step requires systematic assessment of the strengths and weaknesses of each therapeutic option. To this end, we integrate information from clinical studies, case reports, guidelines, and from our own experiences. We list this evidence in a matrix, representing the strengths and weaknesses profile of all therapeutic options for each defined treatment goal. Table 2 shows the SW matrix for all five therapeutic options (A–E), identified as useful to attain the goal of protecting patients with asymptomatic TAAA from rupture.

### 3. Assess individual patient characteristics as opportunities and threats for therapy (OT matrix).

The core of an individualized treatment strategy is the adjustment of medical standards to the characteristics of individual patients [12]; therefore, in this third step of the I‑SWOT analysis, we register critical individual qualities of the individual patient, representing an opportunity or a threat to the therapy. We principally acquire these individual qualities in three relevant dimensions: (i) physical features, e. g. characteristics of aortic pathology, comorbidities, previous medication, allergies, and psychological resilience factors; (ii) social characteristics, such as family, housing, financial resources and other socioeconomic resilience factors; (iii) psychological and intellectual factors, such as the competence to understand and to cooperate, the status of education, mental disorders, such as schizophrenia or depression, attitudes towards life, such as optimism or pessimism, motivation, risk affinity, and psychological and mental resilience factors. Finally, we record the individual values and preferences with potential impact on the therapeutic goal attainment. Finally, we appraise the individual factors of our patients as opportunities or threats according to the treatment goals and list them in an OT matrix.

### 4. Establish a 4‑field I‑SWOT matrix to identify an individualized medical strategy.

**Fig. 5:**
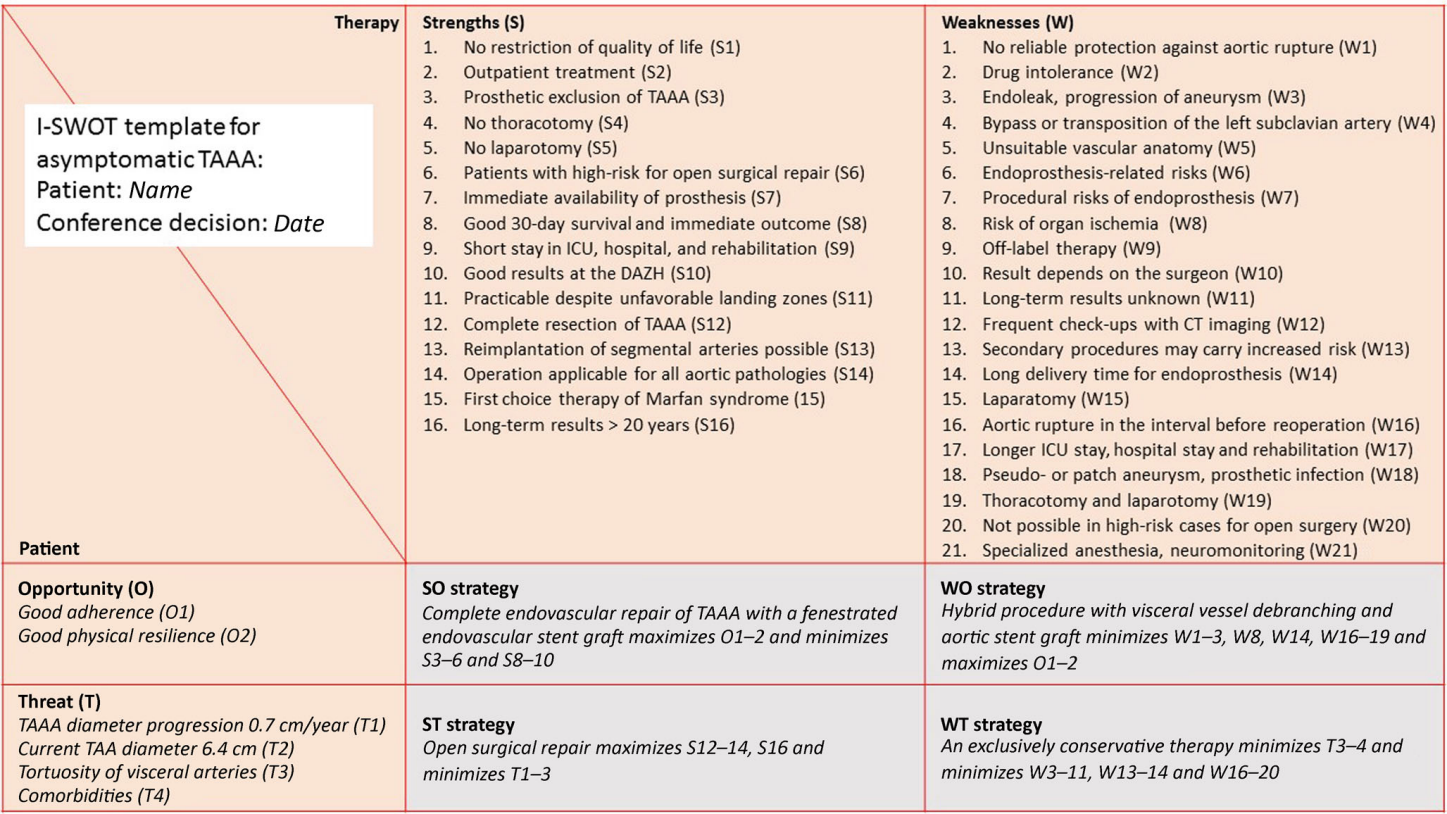
Example of a standard DAHZ template for I‑SWOT for identification of an optimal individual therapeutic strategy for a TAAA

In this final step of I‑SWOT we set up an individualized therapeutic strategy for the patient. To facilitate the strategizing process, we employ I‑SWOT tables which we prefabricate for some of standard therapy goals. In Fig. 5 we show the example of such a fabricated I‑SWOT for the therapeutic goal of protection against TAAA rupture (Fig. 5). In the top row of this I‑SWOT matrix form we list the summary of all possible strengths and weaknesses for the five treatment options (refer to step 2 and Table 1). Unlike the top rows, the first column in I‑SWOT matrix table is not filled out: here, we document the individual characteristics of every patient as opportunities or threats for achieving the therapeutic goal. From the cross-tabulation of standardized therapeutic strengths and weaknesses with the patient’s individual opportunities and threats we are able to set up an individualized medical strategy that maximizes therapeutic success for the individual patient.

## Example for the application of I‑SWOT

We present the example of a well-groomed 70-year-old man, who was working as a general practitioner up to 5 years ago in his own practice. He is still interested in medical affairs and he is highly reliable and adheres to therapy (O1). His current annual check computed tomography angiography (CTA) shows progression of aortic diameter of a TAAA from 5.7 cm in the last year (T1) to 6.4 cm on the current CT scan (T2). The patient has no symptoms. Multidisciplinary review of the CTA images in our aortic conference showed that the visceral vessels could only be supplied at great expense by a completely endovascular procedure (T3). Comorbidities are comprised of untreated arterial hypertension (HTN), a myocardial infarction with a triple aortocoronary bypass (ACB) 4 years ago, and chronic obstructive pulmonary disease (COPD) with FEV1 < 1.0 (T4). He appears to be physically resilient (O2), lives together with his wife and he takes vacation trips to other cities almost three times a year.

In step 1 of the I‑SWOT analysis we identify the goal of the therapy as protection from rupture in asymptomatic TAAA. We have five different and technically possible options to achieve this goal which we principally deemed applicable in our patient. In step 2 we sketch out a strengths-weakness matrix of the five treatment options (Table 1) and fill the strengths-weaknesses profile in the top line of our therapy standard for asymptomatic TAAA (Fig. 5). In step 3 we categorize the individual characteristics of the patient as opportunities (O1–2) and threats (T1–4) for the treatment of TAAA. Finally, we list these opportunities and threats in the I‑SWOT form as O1 and O2 and as T1, T2, T3 and T4, respectively. In the final step we discuss the various therapeutic strategies among colleagues and then together with the patient to integrate the patient’s personal preferences, values and attitudes along with the other opportunities and threats into an individualized strategy. In the following we apply the I‑SWOT matrix to present four possible individualized strategies for the patient:

### SO strategy (maxi-maxi).

A complete endovascular repair of TAAA with a fenestrated endovascular stent graft (option C) requires an experienced surgeon in consideration of the delicate visceral vascular anatomy and it takes a patient with the ability and disposition to measure up and assess risks of an innovative therapy realistically and face these risks for the purpose of maximum achievable medical benefits. The decision for a completely endovascular repair maximizes the patient’s opportunities O1–2 and the therapeutic benefits S3–6 and S8–10.

### WT strategy (mini-mini).

An exclusively conservative therapy (option A) can minimize therapeutic weaknesses W3–11, W13–14, W16–20 by reducing therapeutic measures to a minimum. Simultaneously, this strategy minimizes the patient’s existing threats of the T3–4 for a more aggressive therapeutic approach; however, it is difficult to imagine that our patient will agree with this kind of strategy. Nevertheless, his personal situation may cause at least his temporary commitment, for example, because he needs some time to get over the sudden and unexpected loss of his wife to recover from this stroke of fate.

### WO strategy (mini-maxi).

We may suggest a hybrid procedures with visceral vessel debranching and aortic stent graft (option D). By performing a visceral debranching, we may minimize the therapeutic weaknesses W1–3, W8, W14, and W16–19 of alternative therapeutic options but this method requires an open surgical and an endovascular approach, which is usually done as a 2-stage procedure; therefore, this method requires discipline, capacity for discernment and compliance of the patient which means that we have to rely heavily on the patient’s opportunities O1–2.

### ST strategy (maxi-mini).

An open surgical repair with complete prosthetic graft replacement of TAAA (option E) maximizes therapeutic strengths S12–14 and S16 because it ensures a complete resection of the aneurysm by complete resection of TAAA and replacing the affected aorta with re-implantation of all relevant aortic side branches. This strategy minimizes the patient’s threats T1–3 of extended aortic disease; however, this option requires a patient who is willing to accept the strains and risks of maximum surgery, in order to have the chance to keep the life-threatening disease under control for the rest of his life. It also necessitates a surgeon and a team with the ability and experience to perform such extensive surgery quickly and safely.

## Conclusion

I-SWOT is a method that matches ideal medical standards and guideline recommendations with individual characteristics of a patient. In this way it becomes possible to maximize therapeutic success for the individual patient. I‑SWOT illustrates that there is a choice of four fundamental types of strategy and that the personal disposition of the physician and the patient, expressed as their strengths and weaknesses and as the external threats and opportunities, is important for the choice of strategy. We think that prospective guidelines and evidence-based recommendations should provide strength-weakness matrices for alternative therapy options. I‑SWOT analysis is a flexible tool concerning its grade of formalization so that time exposure can be easily adjusted according to the situation. The application of standardized templates keeps time requirements adequate even if the grade of formalization is high. In our experience, I‑SWOT is an easy to use instrument that helps to prevent technical, ethical and psychological treatment failures. In comparison to a standardized one fits for all application of the medical guidelines, I‑SWOT maximizes the individual therapeutic success by optimizing all three dimensions of treatment goals: effectiveness, compliance, and needs orientation.

## Conclusion for practice

There are two different ways of how to perform guideline-based therapy. One way is the one size fits all approach where all patients receive identical therapy according to guideline standards; however, this way of therapy risks technical, ethical and psychological failure. Therefore, the alternative way of performing guideline-based therapy is to establish an individualized medical strategy. Such individualized medical strategies aim to maximize therapeutic success in the technical, ethical and psychological dimension. Thereby this type of therapy translates guideline recommendations into an effective, norm-compliant, and meeting the patient’s needs type of therapy. I‑SWOT is an easy to use instrument that supports medical decision makers to identify an individualized medical strategy to maximize therapeutic success.
